# Supplementary material for: Pyranoanthocyanins Interfering with the Quorum Sensing of Pseudomonas aeruginosa and Staphylococcus aureus
Source: Int J Mol Sci. 2021 Aug 9;22(16):8559. doi: 10.3390/ijms22168559 (PMC8395250; doi:10.3390/ijms22168559)
Supplement: Supplementary file 1 [file ijms-22-08559-s001.zip › ijms-1317308-supplementary.pdf]

Pyranoanthocyanins Interfering with the Quorum Sensing of *Pseudomonas aeruginosa* and *Staphylococcus aureus*

Patrícia Coelho[1], Joana Oliveira[1], Iva Fernandes[1], Paula Araújo[1], Ana Rita Pereira[1], Paula Gameiro[1], Lucinda J. Bessa[1,*]

[1] LAQV-REQUIMTE, Department of Chemistry and Biochemistry, Faculty of Sciences, University of Porto, 4169-007 Porto; Portugal; patriciaines20@gmail.com (P.C.); jsoliveira@fc.up.pt (J.O.); iva.fernandes@fc.up.pt (I.F.); paula.araujo@fc.up.pt (P.A.); anarita@fc.up.pt (A.R.P); agsantos@fc.up.pt (P.G.)

* Correspondence: lucinda.bessa@fc.up.pt (L.J.B.)

Supplementary Materials

Table S1: Antimicrobial resistance pattern of the multidrug-resistant isolates used in this study.

Isolate	Antimicrobial resistance pattern
Pa3	FEP, GEN, CIP
PA004	CIP, GEN, IPM, PTZ, TOB
SA007*	CIP, CLI, ERI, FOX, GEN, LEV, MOX, OXA
SA011*	CIP, CLI, ERI, LEV, MOX, OXA

CIP: ciprofloxacin; CLI; clindamycin; ERI: erythromycin; FEP: cefepime; FOX: cefoxitin; GEN: gentamicin; IPM: imipenem; LEV: levofloxacin; MOX: moxifloxacin; OXA: oxacillin; PTZ: Piperacillin/tazobactam; TOB: Tobramycin. * These isolates are methicillin-resistant *S. aureus* (MRSA).

Table S2: Primers used in *P. aeruginosa* RT-qPCR analysis.

Gene	Sequence (5′ -> 3′)	Reference
rpoS	Forward: CTCCCCGGGCAACTCCAAAAG	[1]
	Reverse: CGATCATCCGCTTCCGACCAG	
pqsA	Forward: GTTTCTGGTTCCTACCTGCC	
	Reverse: CAGCAGGATCTGGTTGTCGT	
pqsE	Forward: GGTTGAAGGAGGGATCAGCC	
	Reverse: AGTGGTCGTAGTGCTTGTGG	
pqsR	Forward: GATAGCCTGGCGACGATCAA	
	Reverse: CACTGGTTGAAGCGGGAGAT	
lasI	Forward: CAGAACGACATCCAGACGCT	
	Reverse: TCGATGCCGATCTTCAGGTG	
lasR	Forward: AGATCCTGTTCGGCCTGTTG	

	Reverse: GGGTAGTTGCCGACGATGAA	
rhlI	Forward: CAGTTCGACCATCCGCAAAC	
	Reverse: GACGTCCTTGAGCAGGTAGG	
rhlR	Forward: GTTTGCGTAGCGAGATGCAG	
	Reverse: GGCGTAGTAATCGAAGCCCA	

Table S3: Primers used in *S. aureus* RT-qPCR analysis.

Gene	Sequence (5′ -> 3′)	Reference
**16S rRNA**	Forward: CCATAAAGTTGTTCTCAGTT	[2]
	Reverse: CATGTCGATCTACGATTACT	
agrA	Forward: ACGTGGCAGTAATTCAGTGTATGTT	
	Reverse: GGCAATGAGTCTGTGAGATTTTGT	
sarA	Forward: GCTGTATTGACATACATCAGCGAAA	
	Reverse: CGTTGTTTGCTTCAGTGATTCGT	
RNAIII	Forward: GAATTTGTTCACTGTGTCGATAATCCATTT	
	Reverse: GAAGGAGTGATTTCAATGGCACAAGATAT	
ica	Forward: TCGCACTCTTTATTGATAGTCGCTACGAG	
	Reverse: TGCGACAAGAACTACTGCTGCGTTAAT	
hla	Forward: ATGGCTCTATGAAAGCAGCAGA	
	Reverse: AAGGTGAAAACCCTGAAGA	

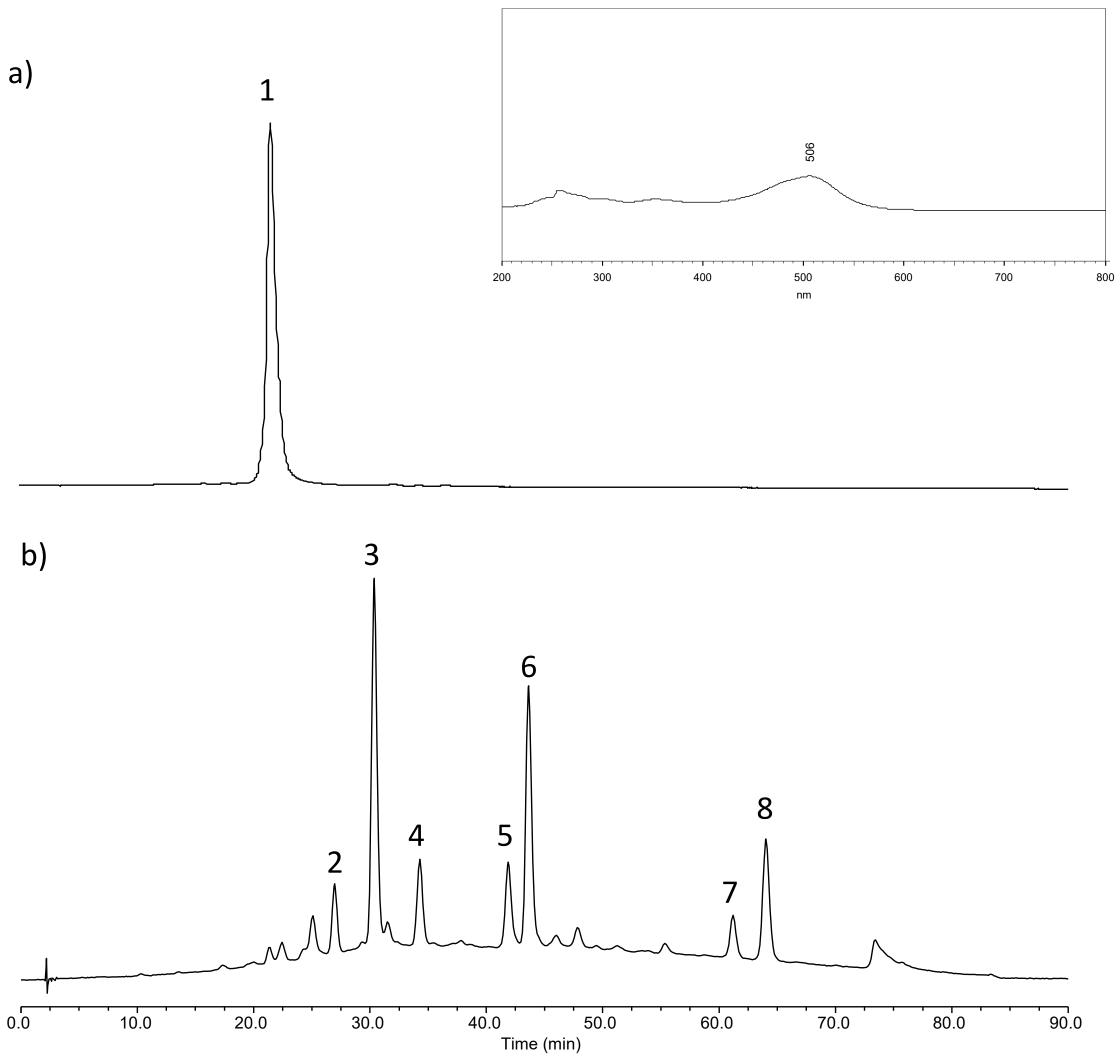

Figure S1. Chromatographic profile of a) 1: carboxypyranocyanidin-3-*O*-glucoside (carboxypyCy-3-glc), UV-vis spectra at the top right and b) red wine carboxypyranoanthocyanins, at 520 nm. 2: Carboxypyranopeonidin-3-glucoside, 3: Carboxypyranomalvidin-3-glucoside; 4: Carboxypyranomalvidin-3-(6″-acetylglucoside); 5: Carboxypyranopeonidin-3-(6″-coumaroylglucoside); 6: Carboxypyranomalvidin-3-(6″-coumaroylglucoside); 7: Peonidin-3-(6″-coumaroylglucoside); 8: Malvidin-3-(6″-coumaroylglucoside).

References

1. Birmes, F.S.; Säring, R.; Hauke, M.C.; Ritzmann, N.H.; Drees, S.L.; Daniel, J.; Treffon, J.; Liebau, E.; Kahl, B.C.; Fetzner, S. Interference with *Pseudomonas aeruginosa* quorum sensing and virulence by the mycobacterial pseudomonas quinolone signal dioxygenase AQDC in combination with the N-Acylhomoserine lactone lactonase QSDA. *Infect Immun* **2019** *87*(10). https://doi.org/10.1128/IAI.00278-19
2. Chen, Y.; Liu, T.; Wang, K.; Hou, C.; Cai, S.; Huang, Y.; Du, Z.; Huang, H.; Kong, J.; Chen, Y. (2016). Baicalein inhibits *Staphylococcus aureus* biofilm formation and the quorum sensing system in vitro. *PLoS One* **2016**, *11*(4):e0153468. https://doi.org/10.1371/journal.pone.0153468
